# Transcutaneous electrical nerve stimulation and solifenacin succinate versus solifenacin succinate alone for treatment of overactive bladder syndrome: A double-blind randomized controlled study

**DOI:** 10.1371/journal.pone.0253040

**Published:** 2021-06-23

**Authors:** Yumeng Zhang, Shaoyong Wang, Shulu Zu, Chanjuan Zhang

**Affiliations:** Department of Urology, The Second Hospital of Shandong University, Jinan, China; University of Alberta, CANADA

## Abstract

**Objective:**

We evaluated a combination of transcutaneous electrical nerve stimulation (TENS) and solifenacin succinate versus solifenacin alone in the treatment of overactive bladder (OAB).

**Methods:**

Ninety-seven female outpatients with OAB were screened for this double-blind randomized controlled study. Eighty-six patients who met our inclusion criteria were divided randomly into two groups. In group A (43 patients), patients received oral solifenacin and “fake” TENS on the foot; in group B (43 patients), patients received oral solifenacin and effective TENS on the foot. Improvements in OAB symptoms were assessed by Overactive Bladder Symptom Score (OABSS), Overactive Bladder Questionnaire (OAB-q), voiding diaries and urodynamic tests. 70 of 86 patients (36 in group A, 34 in group B) completed the 2 months of treatment and 3 months of follow-up.

**Results:**

Statistically, the maximum bladder volume and OAB symptoms of both groups improved significantly after treatment. The improvement in group B was significantly better than that in group A, as indicated by the maximum bladder volume, OAB-q score and voiding diary. Some mild adverse effects were observed, including dry mouth, stomach upset, constipation, muscle pain and local paresthesia.

**Conclusion:**

The combination of TENS and solifenacin was more effective in improving OAB symptoms than solifenacin alone.

## Introduction

According to the International Continence Society, overactive bladder (OAB) is defined as urinary urgency, usually accompanied by frequency and/or nocturia, with or without urgency urinary incontinence, in the absence of obvious urological pathology [1]. OAB can severely influence the quality of life (QoL) and social activities of patients [2].

Guidelines for OAB management set by the American Urological Association suggest that behavioral treatment should be first-line treatment of OAB, including pelvic-floor muscle therapy, bladder training, and fluid management [3]. For patients who do not benefit from first-line treatment of OAB, the standard second-line treatment is oral anti-muscarinic agents and β3 agonists. However, a large proportion of OAB symptoms cannot be relieved by oral pharmacotherapy alone, especially if they are severe [4]. Treatment of severe OAB still remains a very challenging topic for urologists. Many have suggested combination therapy for patients with severe OAB. Several combinations have been reported. Some of them were based on pharmacotherapy, with a single additional therapy [5]; while some of them used two drugs [6, 7].

Electrical nerve stimulation (ENS) shares the same principle as sacral neurostimulation except that it uses the branches of the sacral nerve plexus, rather than the sacral nerve itself. The target of ENS can be anywhere along the path of the tibial nerve or common fibular nerve. It has been noted as a potential therapy for refractory OAB [8, 9]. Multiple clinical trials have shown positive results using ENS [10–14]. However, most trials based on ENS have used percutaneous electrodes. These are invasive, carry the risk of bleeding and infection, and reduce the chance of patient compliance. Transcutaneous electrical nerve stimulation (TENS) is a modification of ENS. It is non-invasive and more convenient for clinical use and uses transcutaneous electrodes to stimulate the sensory nerve endings at the bottom of the foot. These nerves are branches of the tibial nerve, which arises from the sacral nerve plexus. Because of their shallow distribution, they can be stimulated effectively by a non-invasive transcutaneous electrode patch. In treating bladder spasm, TENS achieve a similar effect to that elicited by percutaneous stimulation of the tibial nerve [15, 16].

We designed this clinical trial in order to study the effect of transcutaneous electrical nerve stimulation (TENS) and to find an effective combined treatment for OAB. Our goals were to: (i) evaluate the efficacy of a combination of TENS on the foot and solifenacin; and to (ii) determine if TENS could be a good supplement to therapy using solifenacin in patients with severe OAB symptoms.

## Materials and methods

### Study design

This was a randomized, double-blind, controlled, prospective single-center trial. The study protocol was approved by the ethics committee of the Second Hospital of Shandong University (Shandong, China), and registered at the Chinese Clinical Trial Registry (ChiCTR1800016280). All ongoing and related trials for this intervention have been registered. Written informed consent was provided from each participant.

The primary outcome of this study was the change in maximum bladder capacity measured by urodynamic tests. We used bladder capacity as the primary outcome because it was an objective and quantitative indicator. Secondary outcomes were the change of voiding frequency as recorded by voiding diaries, and improvement of symptoms as measured by the Overactive Bladder Symptom Score (OABSS) and Overactive Bladder Questionnaire (OAB-q).

### Inclusion criteria

The inclusion criteria were: (i) female patient aged 18–75 years; (ii) with OABSS >11; (iii) had not previously received medical treatment;

The exclusion criteria were patients: (i) with severe cardiac diseases or arrhythmia; (ii) contraindications to solifenacin (e.g., urinary retention, gastric retention, uncontrolled angle-closure glaucoma; (iii) who were pregnant or preparing to become pregnant; (iv) who had urinary-tract infection; (v) who had pelvic tumor, lithiasis, genital prolapse, or urinary-tract obstruction.

### Interventions

**Fig 1:**
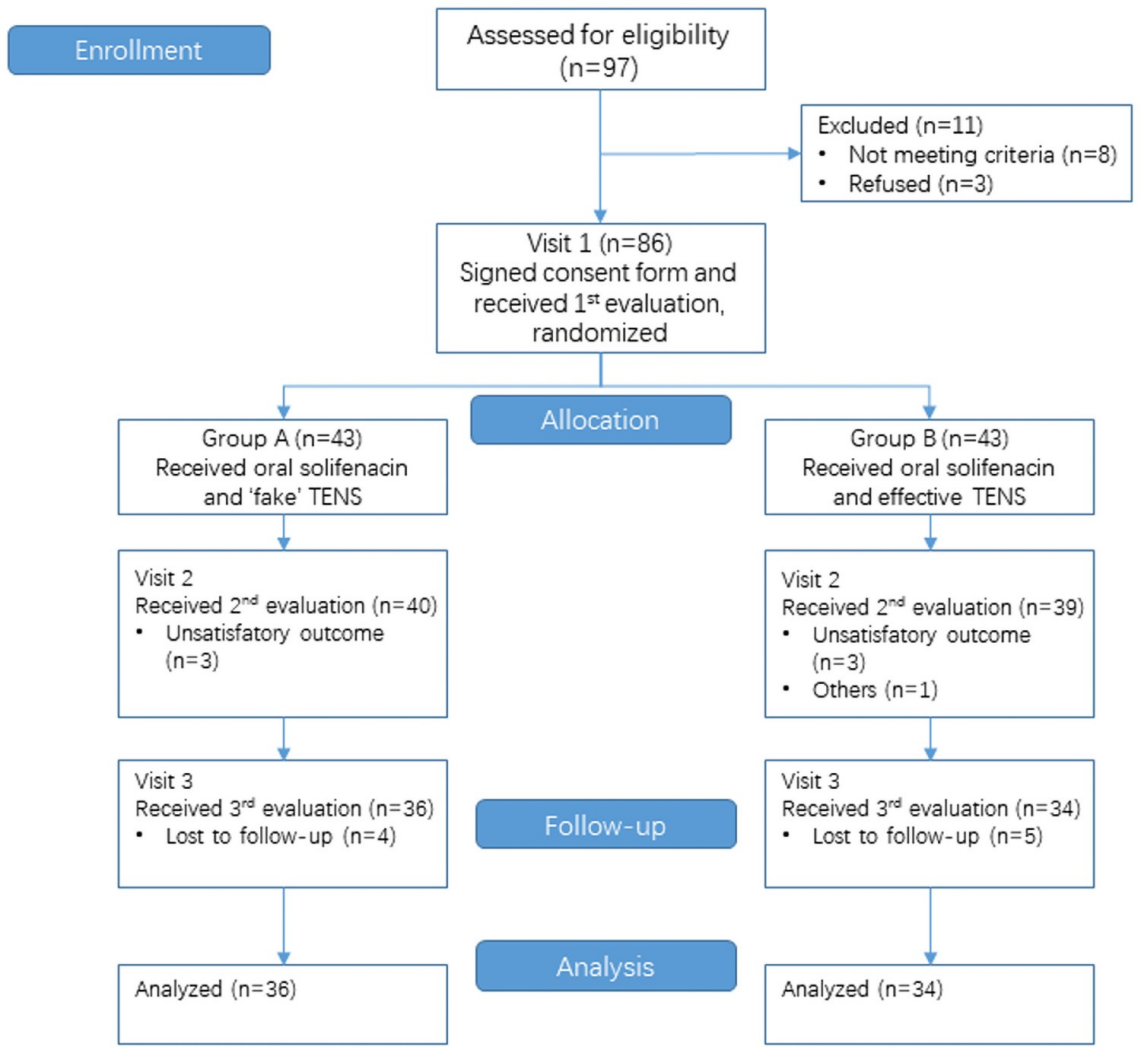
The CONSORT flowchart of this study.

The CONSORT flowchart of this study is shown in Fig 1. From 28 May 2018 to 26 November 2018, 86 female outpatients with OAB symptoms were enrolled. After assessment, patients who met the inclusion criteria were divided randomly into two groups using a random sequence generated by a computer program. The final follow-up was complete on 23 April 2019.

**Fig 2:**
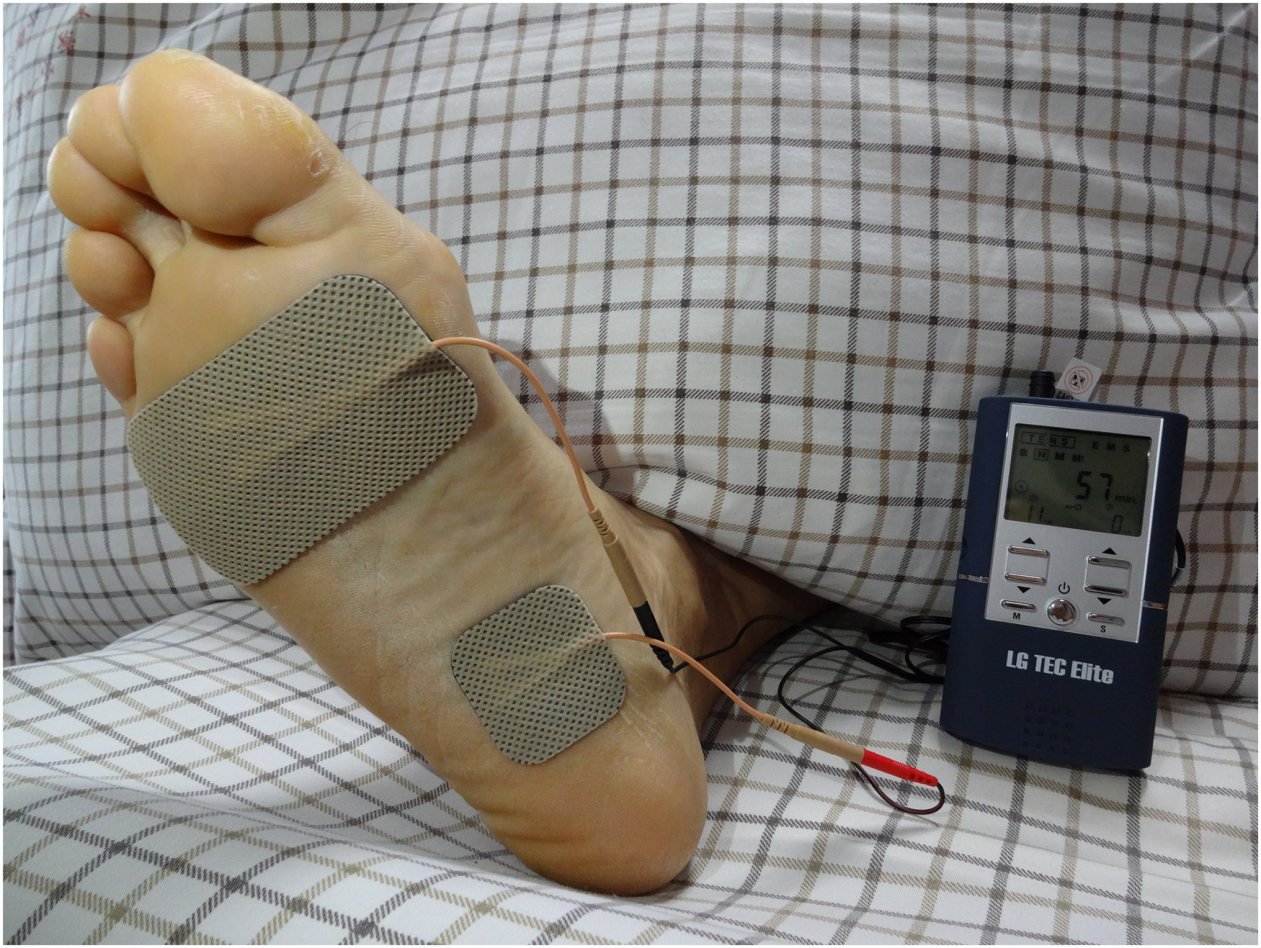
The positions of electrodes on the foot.

For both groups, TENS was conducted with an adhesive skin electrode (LG Med Supply, Cherry Hill, NJ, USA). It was placed at the bottom of the foot (Fig 2) and connected to a transcutaneous electrical nerve stimulator (TEC Elite; LG Med Supply) which provided electrical stimulation (square wave of 5 Hz; pulse width of 0.2 ms).

TENS is done by transcutaneous electrodes, and the stimulation effect is less direct. So in our study, we chose a short-time but more frequent TENS schedule. Patients received over 50 times of TENS treatment in 8 weeks, much more than PTNS (usually 12 times). In group A, patients were given solifenacin succinate (5 mg per day) and received 30 min of “fake TENS” per day. Fake TENS was administered using a “threshold current”, which was the minimum current that could trigger twitching of foot muscles (usually 8–10 mA). In group B, patients received solifenacin succinate (5 mg per day) and 30 min of effective TENS per day. For group B, the stimulation intensity was set to the maximal level comfortable to the patient (usually 60–120 mA). This treatment lasted 8 weeks. TENS was carried out by a technician blinded to patient grouping. After guidance, patients could take away the stimulator and perform TENS at home. The stimulator setting was set and locked by a member of our research team.

### Measurements

The severity of OAB symptoms was evaluated with Overactive Bladder Symptom Score (OABSS). This questionnaire comprises four questions related to daytime voiding, nocturnal voiding, urgency, and leakage. The impact of OAB on patients’ QoL was evaluated with Overactive Bladder Questionnaire short form (OAB-q SF), which comprises symptom bother scale (SBS) and QoL scale. Before treatment, all patients underwent urodynamic tests, and were evaluated with OABSS and OAB-q SF. Patients were also asked to keep voiding diaries for at least three days before the treatment began. Treatment lasted for 8 weeks for both groups. Patients were asked to keep voiding diaries in the final week of treatment. At the end of treatment, all patients were reevaluated again with OABSS, OAB-q SF, and urodynamic tests. Three months after the end of treatment, patients were evaluated the third time with voiding diaries, OABSS and OAB-q SF. Urodynamic tests were conducted by one experienced technician blinded to patient grouping. Evaluation of patient scores was conducted by a member of our research team blinded to patient grouping.

### Statistical analyses

Statistical analyses were undertaken using SPSS 23.0 (IBM, Armonk, NY, USA). P < 0.05 (two-tailed) was considered significant. We used the Student’s *t*-test, chi-square test and ANCOVA to analyze data.

## Results

Within the study period, 97 female patients were diagnosed with OAB in the urology clinic of the Second Hospital of Shandong University. 86 patients met our inclusion criteria and were randomized and divided into two groups. 43 patients received solifenacin succinate and fake TENS (group A) and 43 patients received solifenacin succinate and effective TENS (group B). In total, 70 of 86 patients (36 in group A and 34 in group B) completed 2-month treatment and 3-month follow-up.

Table 1 shows the basic demographic and clinical data of both groups before treatments. The major demographic data of these two groups were not significantly different.

**Table 1:** Age, bladder volume and symptom severity of two groups before treatment.

Group	Age*	Max. bladder volume (ml)	OABSS score	OAB-q SF symptom bother	OAB-q SF quality of life
Fake TENS	57.4(8.9)	137.5	13.3	62.7	39.4
Effective TENS	51.9(17.2)	142.7	13.6	55.2	45.1
p value^+^	0.097	0.527	0.253	0.196	0.317

TENS: transcutaneous electrical nerve stimulation; OABSS: Overactive Bladder Symptom Score; OAB-q SF: Overactive Bladder Questionnaire short form.

* Age is presented as mean(standard deviant)

^+^Between-group comparison. Calculated by independent Student’s *t*-test.

Table 2 shows the improvement of two groups immediately after treatments. Here, all indicators of OAB symptoms improved significantly for both groups. Three months after therapy, OABSS and the voiding frequency of some patients seemed to have worsened slightly, but the difference was not significant. In both groups all p>0.05.

**Table 2:** Bladder volume and symptom severity of the two groups immediately after treatment.

Group	Before Treatment	Immediately after the 3-month treatment	*p-*value*	3 months after the end of the treatment
Max. bladder volume (ml)				
Fake TENS	137.5	152.6	<0.01	NA
Effective TENS	142.7	185.3	<0.01	NA
Daytime urination (times)				
Fake TENS	22.4	19.1	<0.01	17.5
Effective TENS	21.2	16.0	<0.01	16.3
Nighttime urination (times)				
Fake TENS	4.63	3.61	<0.01	3.85
Effective TENS	4.65	3.19	<0.01	3.31
OABSS score^+^				
Fake TENS	13.3	12.1	<0.01	12.1
Effective TENS	13.6	12.2	<0.01	11.3
OAB-q SF symptom bother**				
Fake TENS	62.7	48.3	<0.01	47.1
Effective TENS	55.3	35.2	<0.01	35.4
OAB-q SF quality of life^‡^				
Fake TENS	39.4	53.5	<0.01	55.5
Effective TENS	45.1	63.0	<0.01	62.6

TENS: transcutaneous electrical nerve stimulation; OABSS: Overactive Bladder Symptom Score; OAB-q SF: Overactive Bladder Questionnaire short form.

*In-group comparison before and immediately after treatment. Calculated by paired Student’s *t*-test.

^+^Indicates the severity of OAB symptoms. A higher score denotes more severe symptoms.

**Indicates how much the patient was bothered by OAB. A higher score denoted more botheration.

^‡^Indicates how the symptoms had affected the patient’s life. A higher score denoted higher quality of life.

Table 3 shows the comparison between the two groups immediately after 8-week treatment. Urodynamic tests showed an improvement in the maximum bladder capacity of group B which was significantly better than that of group A (mean increase of 43.4 *vs*. 14.3 mL, *p*< 0.01). The daytime voiding frequency observed in group B was significantly better than that in group A (5.37 *vs*. 3.02, *p*< 0.01). The nighttime voiding frequency in both groups decreased, but the difference was not statistically significant. The between group improvement in OABSS was not statistically significant. The improvement in OAB-q score (SBS and QoL scale) in group B was statistically significantly better than that in group A (22.3 *vs*. 11.8 for SBS, 18.9 *vs*. 13.1 for QoL, *p*< 0.01 and *p* < 0.05 respectively).

**Table 3:** Comparison of the two groups immediately after treatment.

Group	LS mean absolute improvement*	95% CI	*p-*value*
Max. bladder volume (ml)			
Fake TENS	14.3	4.12–24.6	<0.01
Effective TENS	43.4	32.9–53.9	
Daytime urination (times)			
Fake TENS	3.02	2.23–3.82	<0.01
Effective TENS	5.37	4.55–6.18	
Nighttime urination (times)			
Fake TENS	1.03	0.78–1.29	0.062
Effective TENS	1.46	1.20–1.72	
OABSS score			
Fake TENS	1.23	0.87–1.58	0.426
Effective TENS	1.43	1.07–1.80	
OAB-q SF symptom bother			
Fake TENS	11.8	7.82–15.7	<0.01
Effective TENS	22.3	18.3–26.4	
OAB-q SF quality of life			
Fake TENS	13.1	9.55–16.6	0.024
Effective TENS	18.9	15.3–22.5	

LS: least squares; TENS: transcutaneous electrical nerve stimulation; OABSS: Overactive Bladder Symptom Score; OAB-q SF: Overactive Bladder Questionnaire short form; ANCOVA: analysis of covariance.

* LS mean absolute improvements and *p*-value were calculated by ANCOVA. LS mean absolute improvement is the improvement of each variable immediately after treatment from baseline. The ANCOVA model used improvement of each variable as response, treatment group as fixed factor and the corresponding baseline value before treatment as a covariate.

Table 4 shows the adverse effects observed in both groups. The most commonly reported adverse event for both groups was dry mouth. Muscle pain was reported significantly more frequently in group B. Some patients reported that the pain could be as severe as 4–5 out of 10 (Visual Analog Scale). Local numbness was more frequent in group B, but the difference was not statistically significant. However, the pain and numbness often resolved within 2–4 hours, and no patients left the trial because of the pain. Stomach upset and constipation was also observed in a few patients. These complications were mild for most patients, and no patient left the trial because of them.

**Table 4:** Adverse effects observed in both groups.

Group	Adverse effects				
	Dry mouth	Stomach upset	Constipation	Muscle pain	Local numbness
Fake TENS	6	3	1	1	2
Effective TENS	5	2	3	10	6
*p*-value*	0.92	0.95	0.56	<0.01	0.22

TENS: transcutaneous electrical nerve stimulation

*: calculated by chi-square test.

## Discussion

Treatment of refractory OAB poses a considerable challenge for urologists. Oral anti-muscarinics and β3 adrenoreceptor agonists are the first line pharmacological treatment for OAB, but the OAB symptoms of many patients are not satisfactorily relieved by these compounds. Various new treatments have been developed to treat refractory OAB, such as bladder infusion of capsaicin, intradetrusor injection of botulinum toxin and sacral neurostimulation [17]. Though their efficacy has been demonstrated, all of these treatments are invasive and have potentially severe complications [18–20]. Biofeedback requires intra-rectal or intravaginal sensors, as well as frequent outpatient visits. In addition, although initially efficacious, these therapies are associated with a high prevalence of OAB symptom recurrence [21, 22]. A convenient and efficacious therapy for severe OAB is still needed.

Electrical nerve stimulation (ENS) is based on acupuncture used in traditional Chinese medicine. For the mechanics of ENS, it’s assumed that specific stimulation on the tibial nerve can trigger the release of inhibitory neurotransmitters (e.g., gamma-aminobutyric acid, opioid peptides) in the spinal cord [23, 24]. These neurotransmitters can inhibit the sensory and motor nerves of the bladder, and the inhibition remains for hours after the electric stimulation has stopped [25].

TENS uses transcutaneous, instead of percutaneous, electrodes and its target is the sensory nerve endings at the bottom of the foot. It is non-invasive and more convenient for clinical use. One trial has shown that TENS can effectively relieve postoperative bladder spasm [16]. However, studies on the efficacy of TENS on OAB are still lacking.

In this clinical trial, a combination of solifenacin succinate and TENS on the foot was effective in relieving symptoms. On average, the bladder volume increased by 34.6%, y more than when solifenacin succinate was used as monotherapy. In this study, patients had relatively low bladder volume. In China, public knowledge about OAB is still poor and many patients do not seek treatment until their symptoms become nearly unbearable, as a result, many Chinese OAB patients have rather severe symptoms. The results for the OAB-q scale also indicate that combination therapy had a better effect in reducing symptom bother and improving QoL in the combination group. At 3-month follow-up, this combination therapy was not associated with a higher prevalence of symptom exacerbation than that elicited by solifenacin succinate alone. Most symptoms did not worsen after retreatment with solifenacin succinate and TENS had stopped. Some patients’ symptoms worsened after therapy termination, and more patients in group B seemed to be affected, but the overall change in OABSS was not significant. These data demonstrate that combination therapy was associated with a low prevalence of OAB exacerbation in the short-term.

We postulate that TENS on the foot is a promising new treatment for OAB. In the present trial, most patients could carry out TENS unaided in their home. Electrode placement was easy and convenient and patients could operate the simulator after a 30-minute training course. This was a safe method with minimal risk. Many patients reported that their symptoms greatly relieved after the first course of TENS. The most reported adverse effect was muscle pain and local paresthesia, which often resolved within hours. This study showed that the combination of TENS and solifenacin achieved a better effect than that elicited by solifenacin alone, and that this method could merit further investigation.

Our study had three main limitations. First, the duration of follow-up was relatively short. The long-term prevalence of OAB symptom exacerbation of this combination therapy could not be determined clearly. Second, the study cohort was relatively small. Third, patients’ symptoms varied greatly. Because some patients had very severe symptoms, the maximum bladder volume varied from 220 mL to less than 80 mL. Such heterogeneity might have influenced our results. In addition, the main outcome (maximum bladder volume) was not a patient-focused indicator. This might influence the assessment of patients’ reactions.

## Conclusions

Treatment of severe OAB is challenging. The combination of TENS of somatic afferent nerves in the foot and solifenacin succinate was more efficacious than solifenacin succinate alone for OAB patients. This combination therapy is non-invasive, and could be a new way of overcoming OAB. However, further research with larger study population and patient-focused primary outcome is still needed.

## Supporting information

S1 Table Data table. This table listed the raw data we collected in this study.
(XLSX)

S1 Checklist CONSORT checklist. (DOC)

S1 File Study protocol in English and Chinese. (ZIP)
