# Supplementary material for: Transcutaneous electrical nerve stimulation and solifenacin succinate versus solifenacin succinate alone for treatment of overactive bladder syndrome: A double-blind randomized controlled study
Source: PLoS One. 2021 Jun 23;16(6):e0253040. doi: 10.1371/journal.pone.0253040 (PMC8221460; doi:10.1371/journal.pone.0253040)
Supplement: S1 File — (ZIP) [file pone.0253040.s003.zip › Study protocol CHN1.pdf]

山东大学第二医院伦理委员会

批准书

项目名称：经皮电刺激足底神经联合索利那新治疗膀胱过度活动症的随机对照研究

项目负责人：张婵娟　　职称：主管护师　　联系电话：13853142629

所在科室：泌尿外科

合作研究单位：无

研究起止时间：2017年11月-2019年5月

拟申报项目类别及资助金额（或在研项目资金来源及金额）：

院级青年基金

评审意见：

研究项目　经皮电刺激足底神经联合索利那新治疗膀胱过度活动症的随机对照研究

经伦理委员会审查：

研究者的资格、经验符合试验要求；研究方案符合科学性和伦理原则的要求；获得知情同意的方法适当；受试者可能遭受的风险程度与研究预期的受益相比合适。

同意开展该项目的研究。

研究计划书详见附件

2017年10月26日
山东大学第二医院伦理委员会

山东大学第二医院

涉及人的科研项目伦理审查申请表

申报项目名称	经皮电刺激足底神经联合索利那新治疗膀胱过度活动症的随机对照研究
项目负责人及职称	张婵娟 主管护师
研究目的	经皮电刺激(Transcutaneous electrical nerve stimulation, TENS)是利用表面电极刺激及兴奋相应的神经及肌肉,从而达到缓解疼痛的一种简便、无创性的手段,在国外已作为一种有效的缓解疼痛，特别是手术后疼痛的方法。研究提示经皮电刺激可有效舒张膀胱逼尿肌,因而我们推测 TENS 有可能降低或消除膀胱过度活动（OAB）患者的膀胱痉挛症状。本研究以女性 OAB 病人为观察对象,观察经皮刺激脚底感觉传入神经对 OAB 患者症状的改善作用,以期探索一种治疗 OAB 的无创、易操作的新方法。<br>1.疾病负担和治疗现况<br>膀胱过度活动（OAB）是一种以尿急症状为特征的综合征，常伴有尿频和夜尿症状，伴或不伴因膀胱不稳定收缩引起的尿失禁。多发于中老年女性。目前临床上治疗 OAB 的常用药是抗胆碱能药，如托特罗定、索利那新等。但是仍然有大部分 OAB 患者的症状不能仅通过口服药物来控制。对于此类患者，二线治疗方案包括辣椒素膀胱灌注、肉毒杆菌毒素膀胱内注射、生物反馈治疗以及骶神经调节手术等。尽管许多报道已经证实上述方法的有效性，但限于侵入性操作临床应用欠佳。骶神经调节手术作为三线治疗 OAB，也存在手术花费高且操作复杂，一旦失败往往带给患者巨大的身心创伤等缺陷。OAB 患者迫切需要一种有效、无创、易操作的联合 M 受体阻滞剂治疗的新辅助方法。<br>2.本研究目的<br>观察经皮电刺激脚底感觉传入神经联合索利那新治疗 OAB 的效果。
立项依据（简述）	膀胱过度活动（OAB）是一种以尿急症状为特征的综合征，常伴有尿频和夜尿症状，伴或不伴因膀胱不稳定收缩引起的尿失禁。多发于中老年女性。目前临床上治疗 OAB 的一线治疗是行为训练，包括膀胱功能锻炼、盆底肌功能锻炼和饮水管理。当一线治疗无效时，口服 M 受体阻滞剂就成为患者的二线治疗选择。但是仍然有大部分 OAB 患者的症状不能仅通过口服药物来控制。对于此类患者，治疗方案包括辣椒素膀胱灌注、肉毒杆菌毒素膀胱内注射、生物反馈治疗以及骶神经调节手术等。尽管许多报道已经证实上述方法的有效性，但限于侵入性操作临床应用欠佳。骶神经调节手术作为

	三线治疗 OAB，也存在手术花费高且操作复杂，一旦失败往往带给患者巨大的身心创伤等缺陷。OAB 患者迫切需要一种有效、无创、易操作的联合 M 受体阻滞剂治疗的新辅助方法。<br>经皮电刺激 (Transcutaneous electrical nerve stimulation, TENS)是利用表面电极刺激及兴奋相应的神经及肌肉,从而达到缓解疼痛的一种简便、无创性的手段,在国外已作为一种有效的缓解疼痛特别是手术后疼痛的方法。最近在儿童及健康人的膀胱功能研究方面,发现经皮电刺激脚底部神经分布区可使人的膀胱容量增大 50%以上,研究提示经皮电刺激可有效舒张膀胱逼尿肌。因而我们推测，TENS 也可用于 OAB 的治疗。本研究以女性 OAB 病人为观察对象,观察经皮刺激脚底感觉传入神经对 OAB 患者症状的改善作用,以期探索一种治疗 OAB 的无创、易操作的新方法。<br>前期实验结果表明，经皮电刺激足底部的胫神经分布区可有效增加 OAB 患者的膀胱容量及缓解尿急尿频症状。其机制可能与兴奋感觉神经后,通过促进中枢释放抑制性神经递质,从而抑制膀胱的活动及疼痛感受有关。国外大量的动物实验研究证实,以一定的刺激参数(5Hz)电刺激阴部神经或胫神经可促进脊髓抑制性神经递质如阿片肽、氨基丁酸等的释放 ,代谢性谷氨酸受体 3 的激活也可能参与了上述抑制过程。脑脊液内抑制性神经递质的检测及其与电刺激疗效的相关性研究将是我们下一步努力的方向。研究发现电刺激对膀胱功能的调节有明显的后遗效应,本研究发现电刺激的效果以第二天和第三天最明显,这应该与电刺激的后遗效应有关。<br>用经皮电极刺激脚底感觉传入神经治疗 OAB 患者的研究在国内外还无报道。因该方法操作简便、仪器价格低廉(一个刺激器约 500~600 元人民币)且为无创、无副作用、推广性强的效果可靠的方法。如果该研究证实 TENS 刺激脚底感觉传入可有效治疗 OAB，则该方法有望成为除药物治疗之外的 OAB 治疗新手段,也可成为联合治疗的新选择，为广大患者解除精神和经济负担,具有较大的社会效益。<br>本项目的创新之处:(1)首次将无创性的电刺激方法应用到 OAB 患者身上，为将来该方法做为治疗 OAB 的常用手段提供可靠的证据。(2)为药物无效或效果不佳的严重 OAB 患者找到有效的新方法。
受试者类别	门诊病人

受试者例数	1.受试者例数选择依据<br>实验组和对照组遵循电脑生成的随机序列进行分组。此项操作由作是由一位受过临床实验培训的护士应用电脑生成的一个简单的随机分配序列。实验组和对照组均接受索利那新 5mg qd 口服。实验组接受每日一次每次 30 分钟的电刺激。将两个皮肤表面电极固定在足底，10cm×5cm 阴极贴在脚前掌皮肤处，5cm×5cm 阳极贴在足弓与足跟之间的皮肤上。电极连接电刺激发生器上（LG TEC Eliet， LG MedSupply）。选择频率为 5Hz，波宽为 0.2ms 的方形波刺激。首先确定刺激的强度阈值，即强度逐渐增加到引起脚趾抖动的刺激强度即为阈值，以 2∽6 倍于阈值的电流强度（60mA∽110mA）作为干预刺激强度，结合患者耐受力，每日连续刺激 30mim。规律治疗 8 周。由一名经过专门培训的护士，为每一位接受足底电刺激的患者培训并考核使用方法。并将使用流程分发给每一位受试者。建立群打卡模式，监督受试者每日服药及完成有效电刺激足底情况。对照组患者在脚底感觉传入神经贴电极片，给予仅达阈值的电刺激 30 分钟。数据收集两组在同一时间点上。<br>$$n=\frac{2\left(\mu_{\alpha}+\mu_{\beta}\right)^{2}\sigma^{2}}{\delta^{2}}$$<br>$\mu_\alpha=1.64$， $\mu_\beta=1.28$， $\sigma=35$， $\delta=25$<br>N=33.4，考虑到 10%的脱落率，每组至少需要 37 个患者。两组至少需要 74 例。<br>2.研究参加单位和预计纳入参试者例数:<br>山东大学第二医院泌尿外科，预计纳入参试者 75-90 人
受试者选择（入选标准及排除标准）	1.入排标准:<br>选取 2017 年 11 月-2019 年 5 月一所三级甲等综合性医院泌尿外科门诊诊断为 OAB 的患者<br>纳入标准：①女性患者年龄大于 18 岁，小于 75 岁；②无前期药物治疗史；③应用膀胱过度活动症评分问卷表（OABSS）对被试进行评分并诊断为 OAB；④签署知情同意书。<br>排除标准：①有前期治疗；②有下列疾病之一者：盆腔肿瘤、结石、子宫脱垂、尿路感染；③不能完成系统的经皮神经电刺激者；④不能完成后期 3 个月随访者。<br><br>2.如果参加研究将需要做什么?<br>在受试者入选研究前，医生将询问、记录您的病史，用 OABSS 及

	OAB-q 量表对您的症状严重程度进行评分。<br>受试者是合格的纳入者,可自愿参加研究,签署知情同意书。<br>如受试者不愿参加研究,我们将按受试者的意愿施治。<br>若受试者自参加研究,将按以下步骤进行<br>a.简单叙述患者分配流程、各治疗方案(药物:剂量、疗程、使用说明和注意事项、药物生产厂家和批号；采用的治疗和诊断仪器：生产厂家、生产企业许可证、注册证号等)<br>b.患者到医院进行检查和随访的时间、次数、注意事项。<br>需要受试者配合的其他事项：<br>受试者必须按医生指导应用电刺激仪器,并请受试者及时、客观地描述 OAB 相关症状。不可隐瞒症状感受。<br>在研究期间受试者不能使用治疗膀胱痉挛的其它药物。<br>如受试者需要进行其它治疗,请事先与他的医生取得联系。
是否有书面知情同意及履行保密原则	有书面知情同意书及严格履行保密原则。附件如下:<br>知情同意书·知情告知页<br>亲爱的患者：<br>医生已经确诊您为膀胱过度活动症（OAB)。我们将邀请您参加一项临床研究。本研究方案已经得到山东大学第二医院伦理委员会审核,同意进行临床研究。<br>在您决定是否参加这项研究之前,请尽可能仔细阅读以下内容。可以帮助您了解该项研究以及为何要进行这项研究，研究的程序和期限，参加研究后可能给您带来的益处、风险和不适。如果您愿意,您也可和您的亲属、朋友一起讨论,或者请医生给予解释,帮助您做出决定。<br>一、研究背景和研究目的<br>1.疾病负担和治疗现况<br>膀胱过度活动（OAB）是一种以尿急症状为特征的综合征，常伴有尿频和夜尿症状，伴或不伴因膀胱不稳定收缩引起的尿失禁。多发于中老年女性。目前临床上治疗 OAB 的常用药是抗胆碱能药，如托特罗定、索利那新等。但是仍然有大部分 OAB 患者的症状不能仅通过口服药物来控制。对于此类患者，二线治疗方案包括辣椒素膀胱灌注、肉毒杆菌毒素膀胱内注射、生物反馈治疗以及骶神经调节手术等。尽管许多报道已经证实上述方法的有效性，但限于侵入性操作临床应用欠佳。骶神经调节手术作为三线治疗 OAB，也存在手术花费高且操作复杂，一旦失败往往带给患者巨大的身心创伤等缺陷。OAB 患者迫切需要一种有效、无创、易操作的联合 M 受体

	阻滞剂治疗的新辅助方法。<br>2.本研究目的<br>观察经皮电刺激脚底感觉传入神经联合索利那新治疗 OAB 的效果<br>3.研究参加单位和预计纳入参试者例数<br>山东大学第二医院泌尿外科,预计纳入参试着者 75-90 人<br><br>二、哪些人不宜参加研究<br>1.年龄 75 岁以上者；2.有下列疾病之一者：盆腔肿瘤、结石、子宫脱垂、尿路感染；3.之前曾接受过其他治疗者<br>以及正参加其它临床试验的患者，还包括研究人员认为其他原因不适合临床试验者。<br><br>三、如果参加研究将需要做什么?<br>1.在受试者入选研究前,医生将询问、记录您的病史,用 OABSS 及 OAB-q 量表对您的症状严重程度进行评分。<br>受试者是合格的纳入者,可自愿参加研究,签署知情同意书。<br>如受试者不愿参加研究,我们将按受试者的意愿施治。<br>2.若受试者自参加研究,将按以下步骤进行<br>a.简单叙述患者分配流程、各治疗方案(药物:剂量、疗程、使用说明和注意事项、药物生产厂家和批号；采用的治疗和诊断仪器：生产厂家、生产企业许可证、注册证号等)<br>b.患者到医院进行检查和随访的时间、次数、注意事项。<br>需要受试者配合的其他事项：<br>受试者必须按医生指导应用电刺激仪器,并请受试者及时、客观地描述 OAB 相关症状。不可隐瞒症状感受。<br>在研究期间受试者不能使用治疗膀胱痉挛的其它药物。<br>如受试者需要进行其它治疗,请事先与他的医生取得联系。<br><br>四、参加研究可能的受益<br>经皮电刺激(Transcutaneous electrical nerve stimulation, TENS)是利用表面电极刺激及兴奋相应的神经及肌肉,从而达到缓解疼痛的一种简便、无创性的手段,在国外已作为一种有效的缓解疼痛特别是手术后疼痛的方法。最近在儿童及健康人的膀胱功能研究方面发现经皮电刺激脚底部胫神经分布区可使人的膀胱容量增大 50%以上,研究提示经皮电刺激可有效舒张膀胱逼尿肌,因而我们推测 TENS 有可能改善 OAB 症状。<br>尽管已经有证据提示经皮电刺激脚底感觉传入神经有满意的

	疗效,但这并不能保证对您肯定有效。本研究所采用的电刺激方法也不是治疗 OAB 唯一的方法。如经皮电刺激方法对您的病情无效,您可以向医生询问有可能获得的替代治疗方法。<br><br>五、参加研究可能的不良反应、风险和不适、不方便<br>本研究采用的经皮电刺激是国外已经证实了的无创、几乎无副作用的缓解 OAB 症状的新手段。除肌肉酸痛外暂无其他不良反应,在应用过程中需要您每日一次、每次 30 分钟不间断的应用,可能对您在此 30 分钟内的下床活动(如如厕、用餐、散步等)带来不便。<br>如果在研究期间您出现任何不适,或病情发生新的变化,或任何意外情况,不管是否与研究有关,均应及时通知您的医生,他/她将对此作出判断并给与适当的医疗处理。<br>您在研究期间需要按时到医院随访,做一些检查,这些占用您的一些时间,也可能给您造成麻烦或带来不方便。<br><br>六、有关费用<br>经皮电刺激是一项免费项目,包括所用耗材均免费。如出现任何不良反应,医生将尽全力预防和治疗由于本研究可能带来的伤害。如果在临床试验中出现不良事件,医学专家委员会将会鉴定其是否与电刺激或基础治疗有关。申办者将按照我国《药物临床试验质量管理规范》的规定对与试验相关的损害提供治疗的费用及相应的经济补偿。<br>对于您同时合并的其他疾病所需的治疗和检查,将不在免费的范围之内。<br><br>七、个人信息的保密<br>您的医疗记录(研究病历/CRF、化验单等)将完整地保存在您所就诊的医院。医生会将化验和其它检查结果记录在您的病历上。研究者、伦理委员会和药品监督管理部门将被允许查阅您的医疗记录。任何有关本项研究结果的公开报告将不会披露您的个人身份。我们将在法律允许的范围内,尽一切努力保护您个人医疗资料的隐私。<br>按照医学研究伦理,除了个人隐私信息外,试验数据将可供公众查询和共享,查询和共享将只限于基于网络的电子数据库,保证不会泄漏任何个人隐私信息。<br><br>八、怎样获得更多的信息?

	您可以在任何时间提出有关本项研究的任何问题,并得到相应的解答。<br>如果在研究过程中有任何重要的新信息,可能影响您继续参加研究的意愿时,您的医生将会及时通知您。<br><br>九、可以自愿选择参加研究和中途退出研究<br>是否参加研究完全取决于您的意愿。您可以拒绝参加此项研究,或在研究过程中的任何时间退出本研究,这都不会影响您和医生间的关系,都不会影响对您的医疗或有其他方面利益的损失。<br>出于对您的最大利益考虑,医生或研究者可能会在研究过程中随时中止您继续参加本项研究。<br>如果您因为任何原因从研究中退出,您可能被询问有关您使用经皮电刺激的情况。如果医生认为需要,您也可能被要求进行实验室检查和体格检查。<br><br>十、现在该做什么?<br>是否参加本项研究由您自己(和您的家人)决定。<br>在您做出参加研究的决定前,请尽可能向你的医生询问有关问题。<br>感谢您阅读以上材料。如果您决定参加本项研究,请告诉您的医生,他/她会为您安排一切有关研究的事务。请您保留这份资料。<br><br>知情同意书.同意签字页<br>临床研究项目名称：经皮电刺激足底神经联合索利那新治疗膀胱过度活动症的随机对照研究<br>课题承担单位：山东大学第二医院泌尿外科<br>课题协作单位：无<br>同意声明<br>我已经阅读了上述有关本研究的介绍,而且有机会就此项研究与医生讨论并提出问题。我提出的所有问题都得到了满意的答复。<br>我知道参加本研究可能产生的风险和受益。我知晓参加研究是自愿的,我确认已有充足时间对此进行考虑,而且明白:<br>●我可以随时向医生咨询更多的信息。<br>●我可以随时退出本研究,而不会受到歧视或报复,医疗待遇与权益不会受到影响。<br>我同样清楚,如果我中途退出研究,特别是由于药物的原因使我退出研究时,我若将我的病情变化告诉医生,完成相应的体格检

	查和理化检查,这将对整个研究十分有利。<br>如果因病情变化我需要采取任何其他的药物治疗,我会在事先征求医生的意见,或在事后如实告诉医生。<br>我同意药品监督管理部门伦理委员会或申办者代表查阅我的研究资料。<br>我将获得一份经过签名并注明日期的知情同意书副本。<br>最后,我决定同意参加本项研究,并保证尽量遵从医嘱<br><br>患者签名:<br>年 月 日<br>联系电话:<br><br>我确认已向患者解释了本试验的详细情况,包括其权力以及可能的受益和风险,并给其一份签署过的知情同意书副本<br><br>医生签名:<br>年 月 日<br>医生的工作电话:
研究者需要收集的具体材料	电刺激方法:两个皮肤表面电极固定在足底,2x3.5 英寸大的阴极贴在脚前掌处,并尽量覆盖较多的皮肤,2x2 英寸的阳极贴在足弓和足踝之间的足底皮肤。电极连接到 LG TEC 跨皮电刺激发生器,选择刺激频率为 5Hz,波宽为 0.2 毫秒的方形波,首先确定刺激的强度阈值,刺激强度逐渐增加到引起脚趾抖动的强度为阈值,一般选择 2-6 倍于阈值的电流强度(60-110 毫安)作为刺激强度,整个刺激持续稳定,但要保证病人能够在 30 分钟内耐受该强度。<br><br>观察指标:对研究对象在以下方面进行评估:<br>以尿流动力学检查评估患者的膀胱容量,应用《膀胱过度活动症评分问卷表》(OABSS)和《膀胱过度活动症调查问卷简表》(OAB-q)评估患者的症状严重程度及病情对生活的影响,并以排尿日记评估患者的症状。
所收集的受试者材料的检测实验室名称及地点	受试者材料均在山东大学第二医院泌尿外科门诊患者中选取,问卷评估在泌尿外科门诊进行,尿流动力学测试在山东大学第二医院泌尿外科实验室进行

获取材料的方法、地点	患者入组后，先以 OABSS 和 OAB-q 量表进行评估，并记录排尿日记 3 天，之后行尿流动力学检查。<br>规律治疗 8 周后，在治疗的最后一周所有受试者记录排尿日记 3 天。治疗结束后，再次行尿流动力学检查，并再次应用 OABSS 和 OAB-q 量表再次评估。<br>在治疗结束后 3 个月的最后一周，患者再次记录排尿日记 3 天，应用 OABSS 和 OAB-q 量表重新评估。<br>由一名对分组不知情的、经过专门培训的医师对患者进行量表评估并整理结果；另由一名对分组不知情的、专业技师对患者进行尿流动力学检查；由一名研究人员统计整理患者的排尿日记。
研究数据处理（统计方法）	应用 SPSS Statics 统计学软件，应用卡方检验、t 检验及 RMANOVA 检验来分析各组患者的改善情况。以 P<0.05 作为显著性差异的标准。
研究项目起止时间	2017 年 11 月-2019 年 5 月
其他需要说明的事项	我泌尿外科年门诊量约 1 万人次，OAB 是科室的常见病、多发病。小样本数据统计可在短时间内完成。以期尽快完成实验。目前国内外相关文献报道尚未发现,具有非常强的创新性。应尽快扩大样本量，尽早发表文献报道,造福更多人群。<br>科室是国家临床重点专科建设单位,2014 年用于科室建设的专项经费达 500 万元,现拥有省内一流的仪器设备。主要研究者中有一人曾致力于神经泌尿研究 10 年余,属于美国匹斯堡大学(Department of Pharmacology, University of Pittsburgh)的 de Groat 实验室,目前在用的电刺激仪及无损伤双极极贴均从美国购置。本单位 2014 年度获得山东省自然基金联合专项一项,研究课题为:表面电极刺激脚底感觉传人神经治疗膀胱过度活动症的临床研究,前期已由上述课题经费购置电刺激仪 4 台及无损伤双极电极片若干,自行购置专用电池和纸张,项目的前期研究顺利,赞助经费落实到位。其中一名主要研究者于 2014 年 12 月返回美国匹斯堡大学进行部分数据统计及动物实验研究,并与研究室相关负责人员签署帮扶协议,后期的数据统计、动物实验研究及 SCI 论文指导可给予帮助。后期数据处理及动物实验有了 de Groat lab 实验室的大力协助,定能够圆满完成既定目标。

申请科室：泌尿外科　　　　项目负责人：张婵娟

联系电话：13853142629　　　　申请日期：2017.10.17
