# Supplementary material for: Transcutaneous electrical nerve stimulation and solifenacin succinate versus solifenacin succinate alone for treatment of overactive bladder syndrome: A double-blind randomized controlled study
Source: PLoS One. 2021 Jun 23;16(6):e0253040. doi: 10.1371/journal.pone.0253040 (PMC8221460; doi:10.1371/journal.pone.0253040)
Supplement: S1 File — (ZIP) [file pone.0253040.s003.zip › Study protocol EN.pdf]

Ethics Committee of Second Hospital of Shandong University
Certificate of Approval

Study Title: Transcutaneous electrical nerve stimulation and solifenacin versus solifenacin alone in the treatment of overactive bladder syndrome: a randomized controlled study

Person in charge: Chanjuan Zhang, Dept. of Urology

Study Threshold: November 2017 to May 2019

Committee's Comment:
The study entitled *Transcutaneous electrical nerve stimulation and solifenacin versus solifenacin alone in the treatment of overactive bladder syndrome: a randomized controlled study* has been invested by the Ethics Committee of Second Hospital of Shandong University. The researchers are eligible for such trial. The protocol meets scientific and ethical standards. The consent is properly provided. The possible risk of participants is in scale with expected benefits.

The ethics committee has approved this study.

2017.10.26
Ethics Committee of Second Hospital of Shandong University

Second Hospital of Shandong University
Ethics Appliance Form for Clinical Studies

Title	Transcutaneous electrical nerve stimulation and solifenacin versus solifenacin alone in the treatment of overactive bladder syndrome: a randomized controlled study
Person in Charge	Chanjuan Zhang
Propose	Transcutaneous electrical nerve stimulation (TENS) utilizes surface electrodes to stimulate nerve branches. It has already been used as a pain-relief treatment. Previous studies indicated that TENS also had the effect of reducing tension of bladder detrusor, and we believed than it might serve as a new method of treating overactive bladder (OAB). This study would evaluate the combination of TENS and solifenacin versus solifenacin alone in treating female OAB patients, in order to find a simple and non-invasive way to relieve OAB symptoms.<br>1. Current treatment of disease<br>Overactive bladder syndrome (OAB) is defined as urinary urgency, usually accompanied by frequency and nocturia, with or without urgency urinary incontinence. Currently, the first-line treatment of OAB is behavior training, like bladder training, pelvic floor muscle training and fluid managements. For those who failed first-line treatments, the standard second-line treatment of these patients is oral anti-muscarinics. But still a large portion of patients' OAB symptoms could not be relieved solely by oral anti-muscarinics, especially those with severe symptoms. Various new treatments had been developed to treat refractory OAB, like capsaicin bladder infusion, botulinum toxin intradetrusor injection and sacral neuromodulation surgery. Though their efficiency had been proved by multiple reports, all these treatments are invasive in nature and have some severe complications. OAB patients still needs an effective, simple and non-invasive adjuvant treatment.<br>2. Propose of the study<br>To evaluate the combination of TENS and solifenacin versus solifenacin alone in treating female OAB patients, in order to find a simple and non-invasive way to relieve OAB symptoms.
Basic Principles	OAB is defined as urinary urgency, usually accompanied by frequency and nocturia, with or without urgency urinary incontinence. Currently, the first-line treatment of OAB is behavior training, like bladder training, pelvic floor muscle training and fluid managements. For those who failed first-line treatments, the standard second-line treatment of these patients is oral anti-muscarinics. But still a large portion of patients' OAB symptoms could not be relieved solely by oral anti-muscarinics, especially those with severe symptoms. Various new treatments had been developed to treat refractory OAB, like capsaicin bladder infusion, botulinum toxin intradetrusor injection and sacral neuromodulation surgery. Though their efficiency had been proved by multiple reports, all these treatments are invasive in nature and have some severe complications.

	Also, many urologists suggested combination therapy for patients with refractory OAB. Currently, many combinations have been reported, most of them were based on anti-muscarinics, with a single additional therapy. The additional therapy includes herbal medicine, biofeedback, acupuncture and electrical nerve stimulation. Electrical nerve stimulation (ENS) utilizes percutaneous electrodes to stimulate the branch of tibial nerve. Previous studies proved that specific (5Hz) stimulation of tibial nerve can trigger the release of inhibitory neurotransmitter like GABA and opioid peptide in spinal cord. These neurotransmitter can inhibit the unstable contraction of bladder. Multiple clinical trials showed positive results. One trial compared pelvis muscle training, biofeedback and ENS in treating OAB and showed that ENS had the highest relieve rate of 51.4%. Another study conducted a 7-day trial of dorsal genital nerve stimulation on OAB patients, and reported that 81% of patients' urgency symptom significantly relieved. Currently, most trials about ENS used percutaneous electrodes. They are invasive in nature, have additional risk of bleeding and infection, and reduced patients' compliance. Superficial nerves on the bottom of foot are the branches of tibial nerve. Because of their shallow distribution, they can be effectively stimulated by transcutaneous electrode patch, and can achieve similar effect to percutaneous stimulation on tibial nerve. Currently there is no report about the appliance of TENS in OAB patients. We designed this clinical trial to evaluate the efficacy of the combination of transcutaneous electrical stimulation on foot and anti-muscarinics, and to determine if TENS could be a good supplementary to common anti-muscarinics therapy in patients with severe OAB symptoms.<br>TENS has the advantage of non-invasive, simple and economical. It has minimal side effects, and is easy to use. If this study could prove the effectiveness of TENS in treating OAB, TENS might become a new choice of combination therapy, and contribute to drug-refractory OAB patients.<br>New concepts of this study: 1. Utilizes non-invasive TENS in treating OAB; 2.Can provide a new choice for drug-refractory OAB patients.
Subjects	Outpatients
Sample size	Patients would be randomly divided into two groups using computer generated random sequence. This would be done by a trained researcher. TENS would be conducted with adhesive skin electrodes placed on the bottom of foot and connected to an electrical stimulator. Electrical stimulation would be 5Hz square wave, pulse width 0.2ms. In group A, patients were given solifenacin succinate 5mg per day and received 30 minutes of 'fake TENS' per day. The 'fake TENS' was conducted using 'threshold current', which was the minimum current (8-10 mA, using the same stimulator and same pulse setting with group B) that could trigger foot muscle twitching. In group B, patients received 5mg solifenacin per day, and 30 minutes of effective TENS. For group B, stimulation intensity was set to the maximal level (60-120 mA, or 2-6 times of the stimulation threshold) comfortable to the patient. The treatment lasted 8 weeks. Patients' education and TNES treatments were carried out by one of our researchers. The primary outcome of this study would

	be the change of maximum bladder capacity measured by urodynamic tests.<br><br>$$n=\frac{2\ (\mu_{\alpha}+\mu_{\beta})^{2}\sigma^{2}}{\delta^{2}}$$<br>μα=1.64, μβ=1.28, σ=35, δ=25<br>Calculated n is 33.4. Expected lost rate is 10%, so at least 37 patients would be needed for both group.<br>This study would be a single-center randomized controlled study.
Inclusion and Exclusion Criteria	The study threshold is set from November 2017 to May 2019. Test subjects would be outpatients in Second Hospital of Shandong University diagnosed with OAB.<br>The inclusion standards are: 1) female with age between 18 and 75; 2) without previous medical treatment; 3) had no evidence of urinary tract infection; 4) without specific physical diseases (including pelvic tumor, lithiasis, genital prolapse, urinary tract obstruction, etc.).<br>The exclusion standards are: 1) severe cardiac diseases and arrhythmia; 2) contraindications to anti-muscarinics therapy, such as urinary retention, gastric retention, uncontrolled angle-closure glaucoma; 3) pregnancy, or preparation for pregnancy.
Consent and Confidentiality Agreement	Consent form is provided for each patients.<br><br>Clinical Study Consent Form<br><br>Dear Patient:<br>You have been diagnosed of overactive bladder (OAB). We are inviting you to join a clinical study. This study has been approved by the ethics committee of the Second Hospital of Shandong University.<br>Before making your decision, please thoroughly read this form. Its content provided exclusive information about this study, and its purpose, protocol, time threshold, and the possible gain and risk it may cause. If you have any question about its content, inquiry would always be welcomed.<br><br>1. Background<br>Overactive bladder syndrome (OAB) is defined as urinary urgency, usually accompanied by frequency and nocturia, with or without urgency urinary incontinence. Currently, the first-line treatment of OAB is behavior training, like bladder training, pelvic floor muscle training and fluid managements. For those who failed first-line treatments, the standard second-line treatment of these patients is oral anti-muscarinics. But still a large portion of patients' OAB symptoms could not be relieved solely by oral anti-muscarinics, especially those with severe symptoms. Various new treatments had been developed to treat refractory OAB, like capsaicin bladder infusion, botulinum toxin intradetrusor injection and sacral

	neuromodulation surgery. Though their efficiency had been proved by multiple reports, all these treatments are invasive in nature and have some severe complications. OAB patients still needs an effective, simple and non-invasive adjuvant treatment.<br><br>2. Propose<br>To evaluate the combination of TENS and solifenacin versus solifenacin alone in treating female OAB patients, in order to find a simple and non-invasive way to relieve OAB symptoms.<br><br>3. Organizer<br>This study is organized by the department of urology, the Second Hospital of Shandong University. Expected sample size is 75-90 persons.<br><br>4. Inclusion and exclusion criteria<br>The inclusion standards are: 1) female with age between 18 and 75; 2) without previous medical treatment; 3) had no evidence of urinary tract infection; 4) without specific physical diseases (including pelvic tumor, lithiasis, genital prolapse, urinary tract obstruction, etc.).<br>The exclusion standards are: 1) severe cardiac diseases and arrhythmia; 2) contraindications to anti-muscarinics therapy, such as urinary retention, gastric retention, uncontrolled angle-closure glaucoma; 3) pregnancy, or preparation for pregnancy.<br><br>5. What would I be required to do?<br>1). Before entering the study, one doctor will ask about your history, and assess your symptom using printed scales.<br>If you meet the inclusion criteria, you can choose to sign this form, and join this study on your own will.<br>If you are not willing to join this study, we will still perform adequate treatment according to your symptom and your own will.<br>2). If you agreed to join this study:<br>a. You will be informed about the randomization, treatments, examinations (including but not limited to urodynamic test, urinalysis, ultrasound scan) and follow-ups.<br>b. You will be required to follow our time schedule of returning and follow-ups<br>c. You will be required to take the medicine given and use the instruments provided according to our guidance.<br>d. You will be required to tell your doctor about any medicine or medical treatment you would possibly receive during the study threshold.<br>e. You will be required to carefully and honesty finish the given scales and record your own sympotms.<br><br>6. Possible gains

Transcutaneous electrical nerve stimulation (TENS) utilizes surface electrodes to stimulate nerve branches. It has already been used as a pain-relief treatment. Previous studies indicated that TENS also had the effect of reducing tension of bladder detrusor, and we believed than it might serve as a new method of treating overactive bladder (OAB). Animal trials showed that electrical nerve stimulation could increase bladder volume by about 50%, and the effect was long-lasting. Multiple clinical trials also showed positive results.

Though many evidence suggested that TENS could relieve OAB symptom, but it might not work for everybody. The TENS used in this study is not the only treatment for OAB. If TENS is not effective for you, you can inquire your doctor for other treatment at any time.

7. Possible risk and side effects

The TENS used in this study is non-invasive, and has already been proved to be rather safe and have minimal side effect. Main side effect is muscle pain and local numbness, which can often alleviated by itself. You will be required to receive TENS treatment 30 minutes each day. As the treatment should not be interrupted, it might affect your activities like dining, walking and toileting. The medicine(solifenacin) you will be required to taken has side effects of dry mouth, constipation and urinal retention.

Should you have any discomfort during the study, whether related to the study or not, you should inform your doctor at once. He/she would decide further treatments.

You will be required to come back to our hospital and have some examines. This would occupy your time and might cause some inconvenience.

8. Charge

All subjects would not be charged for TENS and medicine(solifenacin) they received. Should any adverse effect happen, your doctor would make his best effort to reduce them. If the adverse effect is proven to be related to the treatments of this study, we will cover the treatment fee, and provide you reasonable compensation.

The examination and treatments for your other disease happened during study period would not be covered.

9. Confidentiality Agreement

Your medical records (including your test results) would be stored in our hospital. Researchers, ethics committee and FDA would be allowed to view your medical records. Any published report about this study would not reveal your personal information. We will protect your personal information and medical records within the allowance of law.

The data generated during this study (without personal information) would be published for open access.

10. Joining and quitting

	You can decide to join or reject this study on your own will. You can also quit this study at any time, without any influence on your treatment.<br>Considering your safety and health, researcher might stop your participation at any time.<br>Should you decide to quit this study, you might still be asked about the effect of treatments on you, or be required to take further examinations.<br><br>11. What to do now<br>You can make your decision (to join this study or not) on your won will.<br>Should you have any question, please inquire your doctor.<br><br>Thank you for reading this consent. If you decide to join this study, please inform your doctor, he will arrange your schedule. One of the copies of this form will be given to you.<br><br>Clinical Study Consent Form<br>Signing Page<br>Study Name: Transcutaneous electrical nerve stimulation and solifenacin versus solifenacin alone in the treatment of overactive bladder syndrome: a randomized controlled study<br>Organizer: Dept. of Urology, Second Hospital of Shandong University<br>Declaration:<br>I have read this consent form and got sufficient information about this clinical study. I know this study's possible risk and gain. I volunteer to join this study. I acknowledge that:<br>I can ask for more information from my doctor at any time;<br>I can quit this study at any time, without influencing the treatment of my disease;<br>If I quit this study, I might still be asked about the effect of treatments on you, or be required to take further examinations;<br>If I was to receive any other medicine or medical treatment during the study threshold, I have to inform my doctor;<br>I agree that my medical record could be viewed by ethics committee or FDA.<br>I will be given a copy of this signed document.<br><br>I agree to join this study and will follow the instructions given by doctors.<br><br>Signature: Date:<br><br>(For doctor)<br>I have exclusively explained the study protocol to the participant, including the possible gain and risk, and have given the participant a copy of this signed document.<br><br>Signature: Date:

Data to be collected	We would collect the following data:<br>1. Maximum bladder capacity, measured by urodynamic test<br>2. Patients' daytime and nighttime frequency, recorded by voiding diaries<br>3. Patients' OABSS and OAB-q scale scores<br><br>Before the start of treatment, all patients received urodynamic test. Patients were also asked to keep voiding diaries for at least three days. For both groups, the treatment lasted 8 weeks. Patients were asked to keep voiding diaries in the last week of treatment. At the end of treatment, all patients were reevaluated with OABSS, OAB-q and urodynamic test. Three months after the end of treatment, patients received were evaluated with voiding diaries, OABSS and OAB-q again. Urodynamic tests were conducted by one experienced technician blind to patients' grouping. Patients' scale evaluations were conducted by one of our researchers blind to patients' grouping. Patients voiding diaries would be analyzed by another researcher blind to patients' grouping.
Place where participants receive tests	Scale assessment would be performed in outpatient department of Second Hospital of Shandong University; Urodynamic test would be performed in the urology lab of Second Hospital of Shandong University.
Statistics	All statistical analyses were performed using SPSS Statistics version 23.0. P values were two-sided and considered statistically significant if less than 0.05. Statistic methods used included Student t-test, chi-square test and RMANOVA.
Study Threshold	November 2017 to May 2019
Other Information	The department of urology of Second Hospital of Shandong University is a state key department. With over 10000 outpatients each year, hopefully we can finish this study within 2 years. Currently our department has already equipped with 4 stimulator, and will have more in the future. Our department has many experienced doctors, technicians and researchers that will ensure the high quality of this study.

Applier: Dr. Chanjuan Zhang, Dept. of urology. Date: 2017.10.17
